# Barriers and facilitators to participation for children and adolescents with disabilities in low- and middle-income countries – A scoping review

**DOI:** 10.4102/ajod.v10i0.771

**Published:** 2021-03-08

**Authors:** Karina Huus, Liezl Schlebusch, Maria Ramaahlo, Alecia Samuels, Ingalill Gimbler Berglund, Shakila Dada

**Affiliations:** 1 Department of Nursing, School of Health and Welfare, Jönköping University, Jönköping, Sweden; 2 CHILD Research Group, School of Health and Welfare, Jönköping University, Jönköping, Sweden; 3 Swedish Institute of Disability Research (SIDR), School of Health and Welfare, Jönköping University, Jönköping, Sweden; 4 Centre for Augmentative and Alternative Communication, Faculty of Humanities, University of Pretoria, Pretoria, South Africa

**Keywords:** adolescents, child, disabilities, barriers, facilitators, participation, low- and middle- income countries

## Abstract

**Background:**

Research has shown that all children and adolescents have the right to participate in their everyday life. However, little is known about what impacts the participation of children and adolescents with disabilities living in low-and middle-income countries.

**Objective:**

The present study undertakes a scoping review of research to synthesise the current literature about barriers and facilitators to participation in everyday life for children and adolescents with disabilities living in low- and middle-income countries.

**Method:**

A scoping review was conducted. The databases Psyc INFO, MEDLINE, CINAHL, Pubmed, ERIC and African Wide information were searched for studies published between 2001 and April 2018. Data was analysed using deductive content analysis. The barriers and facilitators to participation were categorised into personal factors, social factors, environmental factors, and policy and programme factors.

**Result:**

In the end, 17 articles were included for data extraction as they mentioned barriers and facilitators to participation for children and adolescents with disabilities. Most of the reviewed studies reported on barriers to participation. Only one of the studies was performed in a country classified as a low-income country; all other studies were performed in middle-income countries. The results indicate that some factors, especially social factors, could be perceived as both facilitators and barriers to participation.

**Conclusion:**

There is a lack of studies describing barriers and facilitators in low- and middle- income countries. Barriers and facilitators in proximity to the child and family are most frequently described in the literature.

## Introduction

Children and adolescents with disabilities are considered to be a vulnerable population group and as a result require special support and protection (Brown & Guralnick 2012). The United Nations Convention on the Rights of the Child (CRC) is one of several international treaties in which children’s rights are embedded. According to the CRC, all children and adolescents have the right to participation and children’s and adolescent’s own views are considered fundamental. The Global Burden of Disease Report estimated that over 100 million children under the age of 15 years had a moderate or severe disability (Mathers, Fat & Boerma 2004). The majority of children and adolescents with disabilities live in low- and middle-income countries (LMICs). However, the prevalence of children with disabilities is difficult to determine as the prevalence depends on the assessment of the disability.

In recent years, the influence that the environment has on people with disabilities and their lives has been emphasised in conceptual frameworks such as the World Health Organization’s (WHO) The International Classification of Functioning, Disability and Health (ICF) and Child and Youth Version (ICF-CY: WHO, 2007).

Participation is described in the ICF as the involvement in a life situation (WHO, 2007). Involvement in life situations includes the domains of learning and applying knowledge, communication, home life, school life, social life, relationships, leisure and recreation (Maxwell, Alves & Granlund 2012). As the ICF is based on an ecological model of child development and a biopsychosocial perspective, it acknowledges the situational nature of participation, with the environment viewed as a key influencing factor (Anaby et al. 2014). Participation restrictions could appear as a result of the dynamic interaction amongst health conditions, the environment and the person (United Nations, 2007).

Participating in activities of daily life, including both formal and informal leisure activities, is essential for the physical and psychological development of children and adolescents. To take part in activities in the society with other children and adolescents, it is important for children and adolescents with disabilities to grow as individuals and to enjoy life (Anaby et al. 2014; Engel-Yeger et al. 2009). Children and adolescents with disabilities tend to engage in activities, especially outside the family, to a lesser extent than their peers without disabilities (Almqvist & Granlund 2005). From this evidence, it is clear that children and adolescents with disabilities may experience barriers to participation that need to be identified (Brown & Guralnick 2012).

In a systematic review by Shields, Synnot and Barr (2012) that focused on the perceived barriers and facilitators to participation in physical activities for children and adolescents with a disability, they found that the barriers to participation tended to be studied more often than facilitators. Some of the barriers included a lack of knowledge and skills, personal preferences, fear and stigma associated with being disabled, behaviour of parents, infrastructure and programme challenges (lack of transport, facilities, staffing), as well as financial challenges (Shields et al. 2012). On the other hand, facilitators account for the child’s desire to be active and to practise their skills, peer involvement, a supportive family; accessibility to suitable infrastructure, geographic location and information, as well as suitably skilled staff who were able to support participation (Shields et al. 2012). The barriers and facilitators in the Shields et al. (2012) review were categorised into four categories, namely *personal, social, environmental* and *policy and programmes.* Qualitative differences between the perspectives of children or adolescents and caregivers were also noted, with children and adolescents focusing on personal factors rather than familial, social or policy and programme factors like their parents (Shields et al. 2012). It is unclear whether these factors that affect participation of children and adolescents with disabilities would be similar in low and middle-income contexts as most of the studies included in the Shields et al.’s (2012) article were mainly from high-income contexts.

Because the ICF readily acknowledges that participation is influenced by context (Maxwell et al. 2012), it is important that we also understand the factors enabling or preventing the participation of children and adolescents with disabilities in LMICs.

### Rationale

Children and adolescents need to participate in activities of daily life which is important for their physical and psychological development (Anaby et al. 2014; Engel-Yeger et al. 2009). Children and adolescents with disabilities are likely to be less engaged in activities, especially outside the family, compared to typical developed children and adolescents (Almqvist & Granlund 2005). This means that participation restrictions could be identified for children and adolescents with disabilities. Therefore, it is a need for identification of barriers and facilitators for participation in order to enhance participation for these children and adolescents (Brown & Guralnick 2012).

## Methods

A scoping review is one of several types of reviews that have been identified by Grant and Booth (2009), and this is the preferred methodology to be able to map literature on a specific research area, especially when there is a paucity of research in an area. A scoping review is differentiated from a literature review because of the degree of a systematic review in the search. Yet, it does not involve any appraisal of the research evidence as required by a systematic review or meta-analysis (Grant & Booth 2009). In addition, a scoping review usually does not include an appraisal of the quality of the studies and is generally used when there is limited research in a field (Munn et al. 2018). A scoping review involves several steps (Arksey & O’Malley 2005) namely, (1) identifying the research question (2) access to relevant studies (3) selection of studies for detailed analyses (4) analysis of data based on the criteria made by the authors (5) organising and summarising the findings and (6) consultations (which was not done in this study).

### Objectives

The present study undertakes a scoping review of research to synthesise the current literature about barriers and facilitators to participation in everyday life for children and adolescents with disabilities living in LMICs. The barriers and facilitators are discussed relating to personal, social, environmental and policy and programme factors (Shields et al. 2012). The scoping review adheres to the Population, Concept and Context (PCC) format (Schlebusch et al. 2020) with the population referred to children and adolescents with disabilities (up to age 21 years), concept of participation and its family of related constructs (Imms et al. 2017) and context LMICs. Throughout this study, the concept of children and adolescents (up to age 21 years) will be used consistently except for some sentence where children and youth, or young people (0 – 21 years) are used interchangeably. In this study, the 2016 criteria for gross national income according to the Atlas method were used. That is an indicator of income that was developed by the World Bank. Low-income economies are countries with a gross national income per capita of US$1025 or less; lower, middle-income economies are countries with a gross national income per capita between US$1026 and US$4035; upper, middle-income economies are countries with a gross national income per capita between US$4036 and US$12 475, and high-income economies are those with a gross national income per capita of US$ 12 476 or more (World Bank 2016). The population of focus was children and young people (0 – 21 years old) with disabilities and long-term health condition living in LMICs. Disability was defined according to the Convention on the Rights of persons with disabilities, Article 1 as ‘long-term physical, mental, intellectual or sensory impairments that, in interaction with various attitudinal and environmental barriers, hinder full and effective participation in society on an equal basis with others’ (United Nations 2007). The concept of participation is defined by the ICF for children and youth as involvement in a life situation (WHO, 2007). Participation has, according to Imms et al. (2016) two essential components: attendance which is described as ‘being there’ and measured as frequency of attending, and/or the range or diversity of activities, and involvement, the experience of participation whilst attending. Therefore, participation can be defined as attending and being involved in life situations (Imms et al. 2017).

### Protocol and eligibility criteria

This study forms part of a larger study about the participation of children and adolescents with disabilities and/or chronic health conditions, living in LMICs (Schlebusch et al. 2020). The scoping review followed the PCC format. In the study of Schlebusch et al. (2020), a scoping review plan to set parameters for search, screening, extraction and analysis was developed and used. The structure of the study is based on *Preferred Reporting Items for Systematic Reviews and Meta-Analyses* (PRISMA: Tricco et al. 2018). The current study used the data sets from a larger scoping review that Schlebusch et al. (2020) conducted to identify articles that focus on barriers and facilitators to participation of people with disabilities or long-term health conditions in LAMI countries. These articles were further screened to focus on those that reported on barriers and facilitators for participation.

#### Information sources

To be able to find the most relevant articles, search terms were identified through ongoing discussions with the expert panels and in consultation with a health science information specialist to ensure the most relevant yield of articles. A search strategy of combined concepts of interest was applied, using Boolean logic queries: Concept A (children and young people) ‘AND’ Concept B (disability, long-term health condition) ‘AND’ Concept C (participation) ‘AND’ Concept D (LAMICs). Only studies published since June 2001 and before March 2018 were included. The start day of June 2001 was chosen because that was when the ICF was officially endorsed by all 191 member states of the WHO. Even if no language restrictions were applied in the search strategy, only studies published in English were included. Six databases were used for the search: PsycINFO, MEDLINE, CINAHL, PubMed, ERIC and Africa Wide Information. The first electronic search was conducted in December 2016 and an update was done in April 2018 to identify articles meeting the inclusion criteria.

The inclusion criteria for Schlebusch et al. (2020) were: (1) studies including children and adolescents (0 – 21 years old); (2) with disabilities and or long-term health conditions; (3) studies conducted in countries identified as LMICs by the World Bank; (4) studies published in the English language and, for this current paper an additional criterion was added namely, (5) studies describing barriers or facilitators for participation.

#### Selection of sources and evidence

**FIGURE 1:**
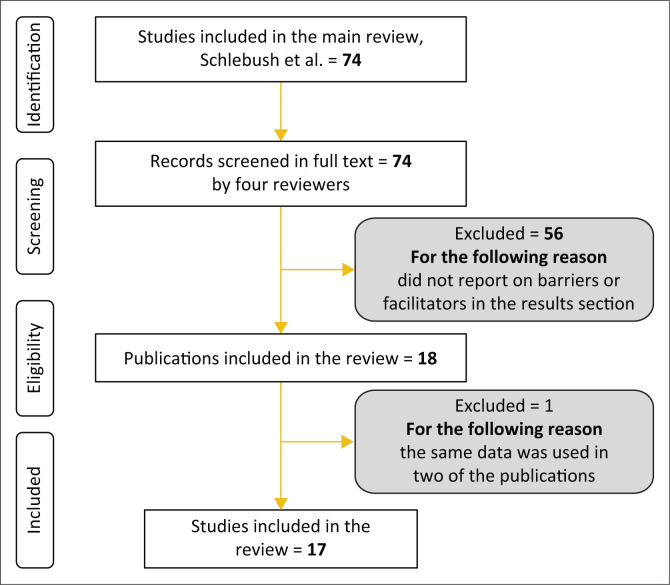
PRISMA flow diagram.

For the study at hand, the total of 74 studies that were included in the review by Schlebusch et al. (2020) was then reviewed for a second time based on the inclusion criteria stated above. Four reviewers (authors) independently reviewed the studies, until 100% agreement between the authors was reached. Amongst the total of 74 studies that were reviewed, 56 were excluded as they did not report data on barriers and facilitators to participation and one was excluded as the same data was used in two different publications. In total, 17 studies that described barriers and facilitators for participation were included (Figure 1). The studies were published between 2009 until 2018. In the study by McConkey et al. 2013 the data collection was done in several countries, of which two countries are LMICs countries, the remaining studies are done in LMICs countries (Table 1).

**TABLE 1:** Table of included studies.

Authors and year	Title	Aim	Study population and method used	Setting	Country	Barriers to participation	Facilitators to participation
Bantjes et al. (2015a)†	Developing programmes to promote participation in sport amongst adolescents with disabilities: perceptions expressed by a group of South African adolescents with cerebral palsy	To understand what a group of adolescents with cerebral palsy living in South Africa perceive to be important components of programmes developed to increase their participation in sport	Adolescents aged 12–18 (*n* = 15) with CP In depth interview. Data analyse interpretative Phenomenological analysis	Special needs school	South Africa	Social barriers Environmental barriers Policy and programme barriers	Environmental facilitators
Bantjes et al. (2015b)†	“There is soccer but we have to watch”: the embodied consequences of theories of inclusion for South African children with cerebral palsy	To explore the lived experiences of children with cerebral palsy and their involvement in physical activity	Adolescents aged 12–18(*n* = 15) with CP, One principal, one teacher and one physiotherapist Ethnographic research Interview Field notes to document observations and informal interactions with staff and pupils	Special needs school	South Africa	Personal barriers Social barriers Environmental barriers	-
Bunning et al. (2014)	Caregiver Perceptions of Children who have Complex communication needs following a home-based intervention using Augmentative and alternative communication in rural Kenya: An intervention note	To investigate preliminary evidence of the impact associated with a home-based, caregiver-implemented intervention employing Augmentative and Alternative Communication (AAC) methods, who have complex communication needs	Children aged 4–12 (*n* = 9) with CP, autism, intellectual disability, hearing impairment. Complex communication needs and their main caregivers Ethnographic research Interview Field notes to document observations and informal interactions with staff and pupils	At the children’s home	Kenya	-	Social facilitators
Columna et al. (2015)	Recreational physical activity experiences amongst Guatemalan families with children with visual impairments	To explore the experiences of Latino families of children with visual impairments living in Guatemala regarding physical recreation	Children aged 5–18 (*n* = 13) with visual impairments Interviews with parents, analysed trough a constant comparative analysis	Sports camp	Guatemala	Personal Environmental barriers	Environmental facilitators
Conchar et al. (2016)	Barriers and facilitators to participation in physical activity: The experiences of a group of South African adolescents with cerebral palsy	To explore the experiences of a group of South African adolescents with cerebral palsy	Adolescents aged 12–18 (*n* = 15) with CP In depth semi-structured interviews	Special needs school	South Africa	Personal Environmental barriers	Personal facilitators Social facilitators Environmental facilitators
Cuhadar and Diken (2011)	Effectiveness of instruction performed through activity schedules on leisure Social facilitators skills of children with Autism. Education and training in autism and developmental disabilities	To investigate the influences of instruction performed through activity schedules on engaging in the schedule skill and fulfilling the activity skills of pre-school children with autism	Pre-school children aged 4–6 (*n* = 3) with autism An intervention that was video recorded	A special education and rehabilitation centre	Turkey		Environmental facilitators
Du et al. (2016)	Relevant Areas of Functioning in People with Adolescent Idiopathic Scoliosis on the International Classification of Functioning, Disability and Health: The Patients’ Perspective	To investigate relevant aspects of functioning and disability, and environmental factors in people with adolescent idiopathic scoliosis according to patients’ self-reports based on the ICF-CY	975 children aged 12–18 with adolescent idiopathic scoliosis Semi-structured interviews	In hospitals	China	Environmental barriers	Social facilitators
Frantz et al. (2011)	Physical activity and sport as a tool to include disabled children in Kenyan schools	To determine the physical activity levels of disabled and non-disabled children at schools in selected provinces in Kenya	Children aged 14–19 (*n* = 599 of these 234 with physical disability and 365 without disability) Self-rating using a questionnaire	Three high Schools that offering inclusive education and three government boarding high schools	Kenya	Personal Social barriers Environmental barriers	Personal facilitators Social facilitators
Glumac et al. (2009)	Guatemalan caregivers’ perceptions of receiving and using wheelchairs donated for their children	To explore the experiences, perceptions, and needs of caregivers receiving wheelchairs donated for non-ambulatory children in a less-resourced country	Children aged 4,1–15,7 (*n* = 14) with CP, Myelomeningocele, Rh Incompatibility, Severe psychomotor retardation Interviews with children (*n* = 5), and with caregivers	Home settings (*n* = 13) Childs therapy setting (*n* = 1)	Guatemala	Environmental barriers	Environmental facilitators
Hansen et al. (2014)	A qualitative study: Barriers and support for participation for children with disabilities	To understand how mothers of children with physical and cognitive disabilities who engaged their children in community-based rehabilitation services perceived and described the level of support they received and the barriers they encountered in terms of their children’s meaningful participation	Children aged 2–21 years (*n* = 11). Physical and cognitive disabilities. Semi-structured interviews with each mother	At the children’s home	Zambia	Social barriers Environmental barriers	Environmental facilitators
Hui et al. (2018)	Gendered experience of inclusive education for children with disabilities in west and east Africa.	Explored the perspectives of community members, police, stakeholders, and children with and without disabilities in order to understand the gendered experience of children with disabilities in west and east Africa	Children in school age (*n* = 30) with disabilities and without disabilities (*n* = 10). Community members 53 (adults) Policy programme stakeholders 19 (adults) Semi structured interviews and focus group discussions	School	Guinea, Sierra Leone, Togo, Niger, Zambia, Malawi	Social barriers Environmental barriers	Environmental facilitators
McConkey et al. (2013)	Promoting social inclusion through unified sports for youths with intellectual disabilities: a five-nation study	To evaluate the outcomes from one sports programme with particular reference to the processes that were perceived to enhance social inclusion	In five countries individual interviews were conducted with five athletes with disabilities, five partners and five coaches, five parents Four head teachers at schools and local politicians Face-to-face interviews based on a standard topic guide Teachers and politician: Group interviews with four teams in country	In a location nearby the sport centre	Germany, Hungary, Poland, Serbia and Ukraine	Social barriers	Social facilitators Environmental facilitators
Memari et al. (2015)	Children with autism spectrum disorder and patterns of participation in daily physical and play activities	To assess the participation of a school-based sample with Autism in physical and daily activities	Children aged 6–15 (*n* = 83) with autism Questionnaire to the parents and to teachers	At the children’s home and school	Iran	Personal Environmental barriers	-
Mudyahoto and Dakwa (2012)	An analysis of the level of participation in sport by learners with disability in inclusive settings	To discuss the extent to which learners with disabilities participate in sport within inclusive settings	Children in school age (*n* = 30) with disabilities like visual impairment, hearing impairment and physical disability Questionnaires with children and teachers (*n* = 20). Interviews with head of institutions and sport masters	School	Zimbabwe	Environmental barriers	-
Mizunoya et al. (2018)	Disability and school attendance in 15 low- and middle-income countries.	To investigate if children with disabilities are systematically more at risk of being out of school in low and middle-income countries and if yes, because of what factors	15 developing countries Used nationally representative dataset from different countries	Register study	Indonesia, Papua New Guinea, Vietnam, Albania, Saint Lucia, West bank Gaza, Bangladesh, India, Maldives, Ethiopia, Malawi, Nigeria, South Africa, Tanzania, Uganda	Social barriers Environmental barriers	-
Nelson et al. (2017)	The meaning of participation for children in Malawi: Insights from children and caregivers	To explore what participation means for children (including those with and without disability) in rural Northern Malawi	Children aged 8–18 with disabilities (*n* = 14), children without disabilities(*n* = 17) Individual interviews focus groups and participatory groups Data from children, professionals and carers	Not specified	Malawi	Social barriers	Social facilitators Environmental facilitators
Vosloo (2009)	The functioning of primary school learners with paraplegia/ paraparesis in mainstream schools in western cape, South Africa.	This study explores the reality of implementing policy guidelines that promote the inclusion of learners with disabilities in mainstream schools	Children aged 6–14 (*n* = 15) with paraplegia/ paraparesis The Craig handicap assessment and reporting technique were used Proxy data from parents and teaching staff	Mainstream schools	South Africa	Environmental barriers Policy and programme barriers	-

ICF-CY, International Classification of Functioning, Disability and Health and Child and Youth Version

† , Combined individually published studies that have the same study participants.

#### Synthesis of results

Specific information relating to the barriers and facilitators was extracted and analysed by two independent reviewers using deductive content analysis, as this is often used when retesting existing data in a new context (Elo & Kyngäs 2008). The analysis in this study was on the four *a priori* themes identified by Shields et al. (2012), namely personal, social, environmental, and policy and programmes. The coding of the data into the categories in the Shields et al. (2012) frameworks was performed by the first and last author, respectively. Discrepancies in the allocation of the codes were discussed until 100% agreement was reached.

### Ethical considerations

This article followed all ethical standards for research without direct contact with human or animal subjects.

## Findings

### Selection and sources of evidence

In total, 17 studies were included in this research (see Figure 1). The findings are categorised into barriers and facilitators, and these parts are further categorised into personal, social, environmental, and policy and programmes (Shields et al. 2012). These 17 studies are described in Table 1, which include the authors, year of publication, title, aim, study population and country of origin for each study, method and the barriers and facilitators of each study.

### Characteristics of sources of evidence and results of individual sources of evidence

#### Barriers to participation in everyday life

A total of 15 studies reported barriers to participation in everyday life of children and adolescents with disabilities in low and middle incomes countries (Bantjes et al. 2015a, 2015b; Columna et al. 2015; Conchar et al. 2016; Du et al. 2016; Frantz et al. 2011; Glumac et al. 2009; Hansen, Siame & Van der Veen 2014; Hui et al. 2018; McConkey et al. 2013; Memari et al. 2015; Mizunoya, Mitra & Yamasaki 2018; Mudyahoto & Dakwa 2012; Nelson et al. 2017; Vosloo 2009). From these 15 studies, 5 reported personal barriers, 8 on social barriers, 13 on environmental barriers and 3 on policy and programme barriers.

**Personal barriers:** Personal barriers included the children’s and the adolescents’ perceptions of their ability, the children and adolescents experienced limitation in body-function hindering their involvement in different activities. The children and adolescents also described fear of getting injured when participating in different activities (Bantjes et al. 2015b; Columna et al. 2015; Conchar et al. 2016; Frantz et al. 2011). The children and adolescents experienced negative emotions with regard to the physical limitations of their bodies, feeling uncomfortable and vulnerable; lacking in sporting spirit. Some children and adolescents did not want to be watched by others whilst participating in physical activities (Conchar et al. 2016).

The children and adolescents were disappointed if they were excluded, felt embarrassed and ashamed at appearing physically inept. The children’s and adolescents’ own intrinsic motivation was also mentioned as a barrier for participation (Frantz et al. 2011; Memari et al. 2015).

**Social barriers:** Social barriers were described as hindrances in the children’s and adolescents’ social lives that prevented them from participating in everyday life. Barriers were reported to occur within the family: some children and adolescents said that they did not feel loved and supported by their immediate family. It could be because mothers found it difficult to deal with a child or adolescent with a disability, and thus the child or adolescent did not receive any assistance from home. In some cases, the father did not feel comfortable when the child or adolescent participated in the family activities (Bantjes et al. 2015b; Frantz et al. 2011; Hansen et al. 2014; Hui et al. 2018; Mizunoya et al. 2018; Nelson et al. 2017).

Children and adolescents reported lacking bonds of friendship in their peer networks and were sometimes bullied or were targets of verbal insults in place of friendship. Some children and adolescents were also harassed by others when participating in physical activities (Bantjes et al. 2015a). On a more general level, some felt that they were regarded as slow and incompetent, and other people made decisions on what they as persons with disabilities should do (McConkey et al. 2013). Concerning the service systems there was a lack of support and care from medical professionals (Nelson et al. 2017).

**Environmental barriers:** Environmental barriers were described as the lack of opportunities and resources within the environment, or the lack of activities for children and adolescents with disabilities to choose from. For example, it could be physical activities that were not adapted to take into account persons with disabilities (Bantjes et al. 2015a). The studies also described a lack of teachers’ knowledge; they did not have any special training in taking care of children and adolescents with disabilities and therefore, many of the school activities were not available to children and adolescents with disabilities (Mizunoya et al. 2018; Mudyahoto & Dakwa 2012). Children and adolescents with disabilities were also excluded from physical activities in school relating to sports and health classes because of lack of suitable equipment and because there were not enough children and adolescents with a similar disability to form a team. Furthermore, there were no schools available in close proximity, which made it impossible for such children and adolescents to attend school (Bantjes et al. 2015a, 2015b; Conchar et al. 2016; Frantz et al. 2011; Hui et al. 2018; Mizunoya et al. 2018).

Barriers in the environment were described as inadequate public transport, poor roads and infrastructure, a busy traffic system and the lack of ramps for wheelchairs. A barrier to accessibility could also be that the students had to take a certain form of transportation home at a fixed time leading to an inability to attend school-based sports activities for the children and adolescents, even if they were available to (Conchar et al. 2016; Glumac et al. 2009; Hansen et al. 2014). Instances where a school did not have space to accommodate additional sport facilities, and had problems accommodating children and adolescents with disability were also cited as barriers (Bantjes et al. 2015b; Conchar et al. 2016; Hansen et al. 2014; Vosloo 2009).

Another reported barrier was that the children and adolescents with a disability were not accepted by others in the community, the school or in their own family. Other people’s attitudes against children and adolescents with disabilities were negative. The studies reported that sometimes peers did not treat children and adolescents with disabilities as equals, or simply did not accept the children and adolescents with disability. Sometimes they even hit the children and adolescents with disabilities (Mizunoya et al. 2018; Vosloo 2009). Some findings alluded that teachers in schools maintained that children and adolescents with disabilities should not take up space for other children and adolescents with a brighter future, and refused to allow a child with a disability into the classroom because they perceived that there was a lack of equipment and lack of knowledge about how to assist children and adolescents with disabilities (Hansen et al. 2014). The children and adolescents with disabilities were also poorly treated (bullied) by some teachers (Hansen et al. 2014; Mudyahoto & Dakwa 2012).

A barrier for children and adolescents with disabilities was that the environment was not adapted to the children’s and adolescents’ needs: for example, an uneven playground (Conchar et al. 2016). The structure of the school day was another barrier because there were no adaptations at the schools to support children and adolescents with disabilities (Bantjes et al. 2015a; Conchar et al. 2016; Mudyahoto & Dakwa 2012). There was also a lack of adapted activities, and those that were adapted were oversimplified and not challenging enough (Bantjes et al. 2015a). Another problem was that the communication style did not adapt to the needs of the children and adolescents with a disability: the of inability to communicate in sign language rendered a situation where the other children and adolescents were not able to communicate (Conchar et al. 2016; Vosloo 2009).

Financial burdens for the family were a constraint as they needed to care for the children and adolescents at home who were unable to earn money (Hansen et al. 2014). It was also expensive to care for a child or adolescent with a disability, and the families often lacked resources to buy proper equipment and hire specialised staff (Bantjes et al. 2015b; Columna et al. 2015). It could also be a financial burden on the family to send the children or adolescents to school, as they could not pay school fees (Memari et al. 2015).

**Policy and programme barriers:** Findings from this review highlighted that there were insufficient policies in place. One example was an inclusion policy that allowed children and adolescents with disabilities to attend mainstream school without the policy stating any requirements for the school to make adjustments needed for equal participation for all children and adolescents (Vosloo 2009). Another study reported on barriers associated with the transportation between school and home (Bantjes et al. 2015a). The policy said that the transport should leave just after school finished, which made it impossible for adolescents with cerebral palsy (CP) to take part in extramural activities that other children and adolescents could enjoy (Bantjes et al. 2015a).

One of the responses also reported a classification system that stratified athletes with similar disabilities into groups, which made the groups too small to compete, along with the lack of space to accommodate additional sporting facilities (Bantjes et al. 2015b).

### Facilitators for participation in everyday life

In this review, 12 studies reported on facilitators for participation in everyday life of children and adolescents with disabilities in LMICs (Bantjes et al. 2015a; Bunning et al. 2014; Columna et al. 2015; Conchar et al. 2016; Cuhadar & Diken 2011; Du et al. 2016; Frantz et al. 2011; Glumac et al. 2009; Hansen et al. 2014; Hui et al. 2018; McConkey et al. 2013; Nelson et al. 2017). From these 12 studies, 2 reported on personal facilitators, 6 on social facilitators and 9 on environmental facilitators.

#### Personal facilitators

Personal facilitators for children’s and adolescents’ participation in everyday life included positive emotions, such as positive perception of the body and body function. It could be factors that improved involvement in physical activity and wanting to control and regulate their body shape and mass. One way of avoiding uncomfortable feelings and maintaining a healthy body was by mastering a physical activity (Conchar et al. 2016). The children and adolescents gained more self-confidence when they had fun and enjoyed themselves. Another facilitator was their experience on learning a new skill (Frantz et al. 2011).

#### Social facilitators

The immediate family could be a facilitator in different ways: for example, if the family had a positive attitude towards the children and adolescents (Du et al. 2016) and the family provided love and support that encouraged the children and adolescents to be active. The children also mentioned that expressions of interest from the family about the children’s and adolescents’ participation in activities inspired them and wished that other people would do the same. It was also important for the children and adolescents to build alliances with their parents (Bunning et al. 2014; Frantz et al. 2011; McConkey et al. 2013; Nelson et al. 2017).

Having friends was another facilitator. To make new friends who had the positive attitude was important. The children and adolescents felt supported and cared for by their friends, and the friends treated them as equals (Conchar et al. 2016; Du et al. 2016; Frantz et al. 2011; McConkey et al. 2013).

Also, support and care from people such as medical professionals was important, along with the opportunities provided to these children and adolescents to participate in various activities. Different augmentative and alternative communication methods could help children and adolescents to participate in social activities (Du et al. 2016; Frantz et al. 2011).

Another facilitator was a positive attitude to the children and adolescents with disability from the general population and the health-care professionals. It is important to build alliances with mainstream schools and for the teachers to have a positive attitude (McConkey et al. 2013).

It is important that coaches worked towards building alliances with sports organisations working with both children and adolescents with disabilities. Improving the ability to move without assistance from others was also important (Conchar et al. 2016).

#### Environmental facilitators

Factors that facilitated availability were dependent on careful planning specific to children and adolescents with disabilities (Columna et al. 2015; Conchar et al. 2016). For example, practical support in the care of children from sources outside of the family made it possible for the children and adolescents to participate in school (Nelson et al. 2017). The photographic scheduler and the teaching process were effective in improving the activity skills of the children and adolescents (Cuhadar & Diken 2011). Team sports with the option to work in groups facilitated participation for children and adolescents with disabilities (Bantjes et al. 2015a).

It was important to have access to suitable facilities in an atmosphere of inclusivity (McConkey et al. 2013). One example would be the access to wheelchairs which made the children and adolescents more independent (Glumac et al. 2009).

The importance of a supportive environment where the children and adolescents felt that they were helped, and that the children and adolescents were accepted by family members, friends and staff had an impact. Friends should not only help them but also be a guide to them (Hui et al. 2018). It is also important to be accepted by the community and neighbours (Bantjes et al. 2015a). Being a resourceful mother will have an impact on how well accepted the children and adolescents are (Hansen et al. 2014).

Careful planning specific to the children and adolescents to ensure that the environment was adapted for the children’s and adolescent’s disability was also important. One example would be when the parents call ahead to ensure that the track they want to take is accessible for a child with visual impairment whilst planning a family trip to the forest (Columna et al. 2015). The external financial aid that the families obtained, such as wheelchairs or economic support made it possible for the children and adolescents to participate (Nelson et al. 2017).

## Discussion

Participation in everyday activities by children and adolescents with disabilities is classified as a fundamental right according to the CRC (United Nations 1989; Viviers & Lombard 2013). The qualitative findings from this literature review indicate that barriers to participation are more frequently reported than facilitators in LMICs. In addition, the findings indicate that some factors, especially social factors can be perceived as both facilitators as well as barriers.

The most frequently mentioned barriers were social barriers. Such barriers included children not being accepted as they were and the prejudices of others in not wanting them to participate.

In an article by Huus et al. (2016) about how primary caregivers in a low-income setting perceived children’s rights, it was found that participation was the least commonly mentioned right by the primary caregivers. However, whether the same pattern can be seen in high-income settings needs to be investigated. The finding that social barriers are most frequently mentioned is probably universal, independent of socioeconomic standards in the country of study. Rather than indicating a qualitatively different pattern in LMIC countries, the findings confirm earlier studies in high-income settings (Law et al. 2007).

Social barriers were found in the everyday activities of children and adolescents with disabilities in their immediate family, amongst the children’s and adolescents’ friends, and by professionals. Most often it was the attitudes of others that influenced whether the children and adolescents with disabilities participated or not. So even if the children and adolescents were in attendance, they were not actively involved (or participated) in the activity (Bantjes et al. 2015b; Frantz et al. 2011). Often, people around the child and adolescent were not aware of the children’s rights under the UN convention. Some people had a perception that the children could not participate and they found it difficult to conceive what kind of changes were needed to make it possible for the children and adolescents with disability to participate. An example of a factor described as both a facilitator and a barrier is in the attitudes of family and friends. It is encouraging when children and adolescents with disabilities experienced the family support in enabling them to participate, and friends treated them as equal partners. The opposite was when there was a lack of encouragement for participation of children and adolescents with disabilities from family and friends.

Only barriers were reported in the policy and programme category. This is contradictory to Shields et al. (2012) where the majority of barriers and facilitators towards participation of children and adolescents with disabilities were found under policy and programmes. One aspect that appears in this study as a barrier was the inclusion policy that allowed children and adolescents with disabilities to attend mainstream schools, without making adjustments needed for equal participation of all children. In a Swedish study by Olsson et al. (2020) (high-income country) it was found that when children and adolescents with disabilities were integrated into mainstream schools, they were less likely to receive disability-related services from rehabilitation services. The teachers in mainstream schools did not know about available services and could not help the primary caregivers in contacting the concerned authorities. Another policy and programme barrier described was problems with transportation between the school and home. This is also shown in a literature review by Lygnegård et al. (2013) focusing on the needs of children with disabilities living in poverty-ridden settings. This review also showed that transportation is a common issue; particularly how it is organised.

Participation in sport at an early age is good for the children’s and adolescents’ development, fostering a healthy lifestyle. The benefits of an active lifestyle could improve feelings of inclusion and the children’s and adolescents’ self -esteem (Wilson & Clayton 2010). Children and adolescents with disabilities also need to have physical activity for the same reasons. However, in this study physical activity was something that troubled the children and adolescents with disabilities because they were often divided into groups depending on the kind of disability they had, and when the groups were too small to constitute a complete team their participation was denied (Wilson & Clayton 2010). The question arises whether the teams must compulsorily have the same numbers of players. The inclusiveness of the team may be more important than the size, as described by the children and adolescents in a study by Spencer Cavaliere and Watkinson (2010). The children and adolescents indicated that they would feel more inclined to engage in a physical activity if features relating to gaining entry like any other legitimate participant were provided and also, having friends (Spencer-Cavaliere & Watkinson 2010).

## Limitations of the study and directions for future research

There are notable limitations of this scoping review: the inclusion criteria of peer-reviewed articles are a potential publication bias. It is more likely that significant results are published than non-significant or negative results. In this study, an overview of the material is presented (Schlebusch et al. 2020). A lot of studies describe participation, but only a few describe barriers and facilitators for participation of children and adolescents with disabilities. Barriers and facilities for participation are often briefly described in an article, and there are almost no studies focusing particularly on this subject.

Few studies were conducted in low income countries with most studies being performed in middle- or high-income countries. It is important that future research focuses on barriers and facilitators in low-income countries in order to map the requirements and to tailor the interventions to enhance the participation of children and adolescents with disabilities.

## Conclusion

There is a lack of studies describing barriers and facilitators to the participation of children and adolescents with disabilities in LMIC. The findings from the current study indicate that barriers are more frequently described, and they are also described in more depth. Barriers and facilitators in the proximity of the child or adolescents and the family, as within the categories of personal and social barriers and facilitators are most frequently described. In contrast to the studies in high income countries, barriers to policy and programmes, more distant to the child or adolescents and the family, were less frequently described.
